# Supplementary figures and images for: Epigallocatechin Gallate Modulates Microglia Phenotype to Suppress Pro-inflammatory Signalling Cues and Inhibit Phagocytosis
Source: Mol Neurobiol. 2023 Dec 14;61(7):4441–53. doi: 10.1007/s12035-023-03845-3 (PMC11236853; doi:10.1007/s12035-023-03845-3)

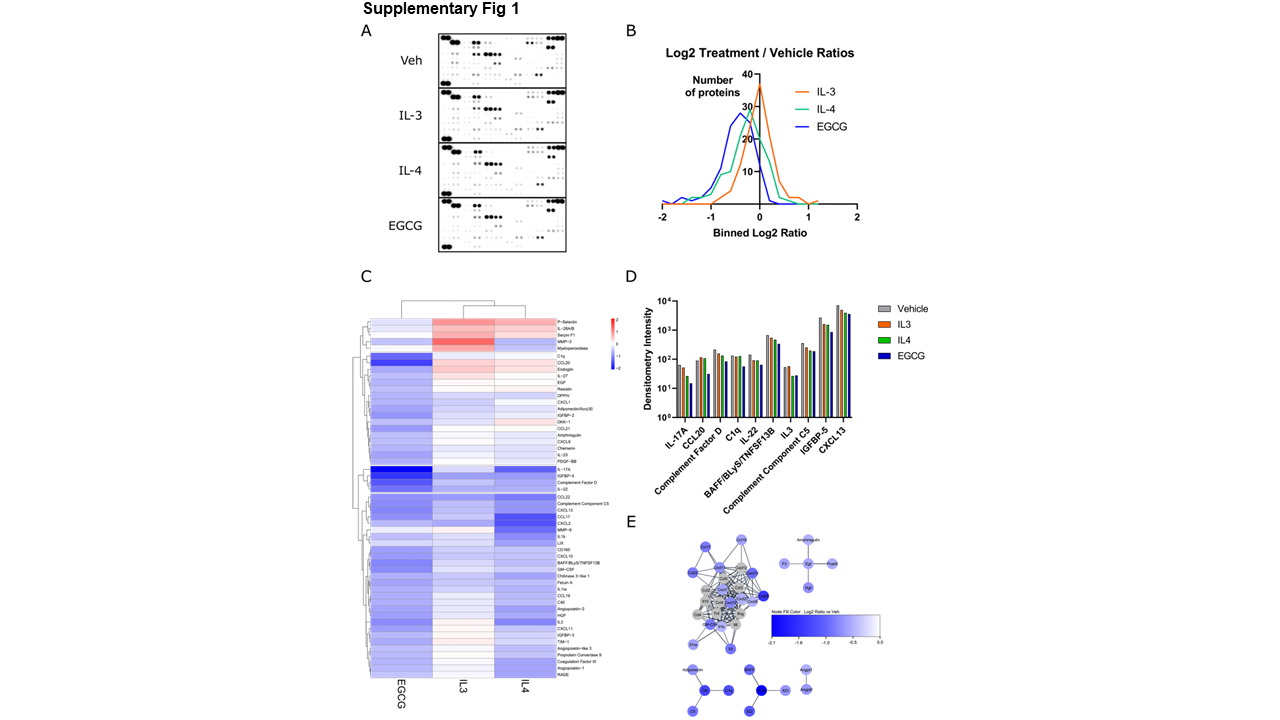

Supplement: Supplementary file 1 — Supplementary file1 Supplementary Figure 1. EGCG reduces inflammatory cytokine release from microglia. (A) Dot blots of 111 different cytokines (spotted in duplicate) of the Proteome Profiler Mouse XL Cytokine Array exposed to conditioned media from microglia treated with EGCG (1µM) or IL-3 (20ng/ml) or IL-4 (20ng/ml) for 24h. (B) Log2 ratios of densitometric readings for all 111 cytokines from (A) for each treatment relative to Vehicle. (C) Heatmap and hierarchical clustering of cytokines showing an absolute log2 Treatment/Vehicle ratio > 0.5 in any of the treatment conditions. Colour indicates log2 Treatment/Vehicle ratio. (D) Densitometry values of the top 10 cytokines modified by EGCG relative to Vehicle. (E) STRING network analysis of functional protein associations between the top EGCG modified cytokines. Node colour indicates Log2 EGCG/Vehicle densitometry ratio for each cytokine (grey nodes are functionally associated proteins that were not changed/tested in the Profiler Array). Edges represent predicted protein associations with stringdb confidence scores > 0.8 (mapped to line width). (TIF 195 KB) [file 12035_2023_3845_MOESM1_ESM.tif]
